# Associations of excessive screen time and early screen exposure with health-related quality of life and behavioral problems among children attending preschools

**DOI:** 10.1186/s12889-022-14910-2

**Published:** 2022-12-27

**Authors:** Hongyu Xiang, Li Lin, Weiqing Chen, Chunrong Li, Xinxia Liu, Jinghua Li, Yan Ren, Vivian Yawei Guo

**Affiliations:** 1 Department of Epidemiology, School of Public Health, Sun Yat-sen University, Guangzhou, 510080 Guangdong China; 2 Chengdu Women’s and Children’s Central Hospital, School of Medicine, University of Electronic Science and Technology of China, Chengdu, 611731 Sichuan China; 3 Zhongshan Third People’s Hospital, Nanlang Town, Zhongshan, 528451 Guangdong China; 4 Department of Biostatistics, School of Public Health, Sun Yat-sen University, Guangzhou, 510080 Guangdong China

**Keywords:** Screen time, Early screen exposure, Health-related quality of life, Behavioral problems

## Abstract

**Background:**

Both excessive screen time and early screen exposure have been linked to children’s health outcomes, but few studies considered these two exposures simultaneously. The aim of this study was to explore the independent and interactive associations of excessive screen time and early screen exposure with health-related quality of life (HRQOL) and behavioral problems among Chinese children attending preschools.

**Methods:**

A cross-sectional study of 4985 children aged between 3 and 6 years was conducted in Chengdu, China. Each parent has finished an online questionnaire regarding their children’s screen use, HRQOL, and behavioral problems. Children with screen time over 1 h/day were considered as having excessive screen time. Early screen exposure was defined if the children had started using screen-based media before the age of 2 years. HRQOL was assessed by the Pediatric Quality of Life Inventory version 4.0 (PedsQL 4.0), while behavioral problems were confirmed with the 48-item Conners’ Parent Rating Scale (CPRS-48).

**Results:**

Of the 4985 children (2593 boys and 2392 girls) included, the mean age was 4.6 (SD: 1.0) years. After adjustment for confounders and early screen exposure, excessive screen time was significantly associated with worse HRQOL scores in all dimensions and summary scales, as well as each type of behavioral problems (all *p* values < 0.05). We also found that compared to children with later initiation of screen exposure, those with screen use before the age of 2 years had significantly lower emotional functioning score (*β*: − 2.13, 95%CI: − 3.17, − 1.09) and psychosocial health summary score (*β*: − 0.82, 95%CI: − 1.54, − 0.10) of HRQOL, as well as higher risks of conduct problems, learning problems, psychosomatic problems, impulsive-hyperactive, and hyperactivity index, which were independent of excessive screen use. Furthermore, there were significant interactive effects of excessive screen time and early screen exposure on emotional functioning domain of HRQOL scores and conduct problems.

**Conclusion:**

Excessive screen time and early screen exposure are two independent and interactive factors to children’s HRQOL and behavioral problems. Our findings support current guidelines to limit screen exposure in children. Appropriate screen use may represent an important intervention target to improve children’s HRQOL and reduce their behavioral problems.

**Supplementary Information:**

The online version contains supplementary material available at 10.1186/s12889-022-14910-2.

## Background

Screen-based media has become a central part of daily life in the current young generations. According to recommendations by the World Health Organization (WHO), screen time should be avoided for children less than 2 years old, and no more than 1 hour per day for children aged between 2 and 5 years [1]. In China, the 2021 Physical Activity Guidelines even have stricter criteria [2]. It recommended no screen-based media for children aged between 0 and 2 years, less than 1 hour of screen time per day for children aged between 3 and 5 years, and less than 2 hours of screen time per day for those aged between 6 and 17 years [2]. Despite above-mentioned guidelines, excessive screen time is still prevalent among preschool children in China [3, 4]. For example, a cross-sectional study of Chinese children aged between 3 and 6 years has found that around 55% of the children spent > 1 hour per day on screen-based media [3]. Another cross-sectional study with 29,461 preschool children in China has also shown that approximately 62% of the children had daily screen time ≥ 1 hour, and 60% of them had started using screen-based media before the age of 2 years [4].

A growing body of evidence suggests that excessive screen time and early screen exposure have detrimental impacts on children [5–9]. For example, a multi-country cohort study has revealed a link between excessive screen time and an increased risk of overweight or obesity in children aged between 2 and 11 years [5]. Likewise, other studies have demonstrated significant associations of excessive screen time with shorter sleep duration, poor sleep quality, and poor cognitive development in children [6–8]. Furthermore, early screen exposure has also been found to be a risk factor for language delay in children [9].

In addition to above-mentioned health issues, excessive screen time and early screen exposure could also affect children’s behaviors and health-related quality of life (HRQOL) [10–13]. A cross-sectional study of Chinese children aged between 3 and 6 years has found that daily screen time over 2 hours was associated with increased risks of behavioral problems [10]. Another longitudinal study in Australia has further shown that adolescents with excessive screen time had significantly lower scores in multiple domains of HRQOL compared to those with moderate screen time exposure [11]. In regard to the detrimental impact of early screen exposure on children, a cross-sectional study has reported significant associations between early screen exposure and emotional/behavioral difficulties among Singapore children aged between 2 and 5 years [12]. A cross-sectional study of Chinese preschool children has also shown that the risk of autistic-like behaviors was 1.90 times higher in children with screen exposure before 3 years old, compared to their counterparts who had never been exposed to screen-based media [13].

Although HRQOL is an important indicator that reflects a person’s physical, psychological, and social well-being [14], its associations with excessive screen time and early screen exposure were rarely discussed in children attending preschools. Furthermore, the independent and interactive impact of excessive screen time and early screen exposure on children’s HRQOL and behavioral problems also warrants further investigations. Therefore, in this cross-sectional study, we aimed to first explore the independent associations of excessive screen time and early screen exposure with HRQOL scores and behavioral problems among children attending preschools in Chengdu, China. Then, its interactive impact on children’s outcomes was also evaluated.

## Methods

### Study design and population

**Fig. 1:**
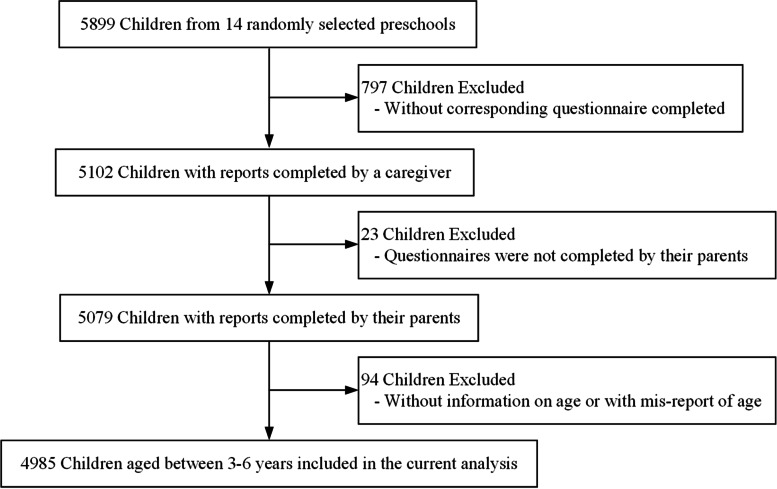
Flowchart of participants selection

This cross-sectional study was conducted from May to July 2021 in Chengdu, a mega-city located in western China. It is comprised of 12 urban districts, 5 county-level cities, and 3 counties. To select representative children attending preschools, a multistage sampling strategy was used. In the first stage, 4 urban districts, 2 county-level cities, and 1 county were randomly selected. In the second stage, two preschools were further randomly chosen from each selected area. A total of 14 preschools were finally included. All the children and their parents in the randomly selected preschools were invited to join this study. During the recruitment period, caregivers of 5102 children have finished an online questionnaire (response rate: 86.5%). We have further excluded 23 children with questionnaires completed by other caregivers, rather than their parents, and 94 children with missing data on age or with mis-report of age, as the eligible age of a child for enrollment is between 3 and 6 years in these 14 participating preschools. Finally, a total of 4985 children aged between 3 and 6 years were included in the current analysis (Fig. 1).

This study was approved by the Ethics Committee of School of Public Health, Sun Yat-sen University (Reference number: 2021[116]). Each parent has signed an informed consent before attending this study.

### Excessive screen time and early screen exposure

Children’s daily screen time was reported by their parents using the following questions: “On average, how many hours per day does your child usually spend on the computer, tablet, mobile phone, TV, or e-reader on a typical weekday and weekend day, respectively?”. The average daily screen time was calculated as (5 × daily screen time on weekdays + 2 × daily screen time on weekends) /7. We further categorized daily screen time into the moderate screen time group (≤ 1 h/day) and the excessive screen time group (> 1 h/day), according to previous guidelines [1] and studies [3, 15].

Furthermore, parents reported the specific age when their child first had electronic screen exposure by the question: “How old is your child when he/she first had access to electronic devices, including computer, tablet, mobile phone, TV, or e-reader?”. Early screen exposure was confirmed if the children had started using screen-based media before the age of 2 years according to the WHO guideline; otherwise, he or she was considered as having later initiation of screen exposure [1].

### Measurement of children’s HRQOL and behavioral problems

Children’s HRQOL was measured using the Pediatric Quality of Life Inventory version 4.0 (PedsQL 4.0) [16], which has been demonstrated to be reliable and valid in Chinese children [17, 18]. The instrument has 21 items for children aged between 2 and 4 years, and 23 items for children aged between 5 and 7 years. Both versions have four dimensions, including emotional functioning (5 items), physical functioning (8 items), social functioning (5 items), and school functioning (3 items for 2–4 years old children, and 5 items for 5–7 years old children). To calculate HRQOL scores, each item was first rated based on a five-point Likert scale (0 = never a problem, 1 = almost never a problem, 2 = sometimes a problem, 3 = often a problem, and 4 = almost always a problem). Then, the items were reverse-scored and linearly transformed to a 0–100 scale, so that higher scores indicated better HRQOL. A psychosocial health summary score was calculated as the average scores of the emotional functioning, social functioning, and school functioning dimensions. A total scale score of the overall HRQOL was further calculated as the average scores of all items in the four dimensions.

Children’s behavioral problems were assessed by the 48-item Conners’ Parent Rating Scale (CPRS-48) [19], a validated questionnaire to evaluate behavioral problems in Chinese children aged between 3 and 18 years [20]. It measures six different types of behavioral problems, including conduct problems (12 items), learning problems (4 items), psychosomatic problems (5 items), impulsive-hyperactive (4 items), anxiety (4 items), and hyperactivity index (10 items). Each item was scored based on a four-point Likert scale, i.e., 0 = never, 1 = occasionally, 2 = often, and 3 = very often. The final score of each type of behavioral problem was calculated by averaging the scores of the corresponding items. For each dimension, the abnormality was defined as a score ≥ 90th percentile based on the norm distribution in China [20].

### Covariates

#### Children

Children’s information on age, gender, status of single child, and primary caregiver were reported by their parents. The status of single child was classified as having only one child in the family (yes) or having two or more children in the family (no). The primary caregivers of children were classified into three groups as mothers, fathers, and others (grandparents or other people).

#### Parent

Parents self-reported their age, marital status, education level, monthly per-capita income, family harmony level, parenting styles, and negative emotional states. Marital status was categorized as currently married or unmarried. The latter included single, divorced, separated, and widowed. Education level was grouped into four categories: (1) junior high school or below, (2) senior high school, (3) bachelor’s degree, and (4) master’s degree or above. Monthly per-capita income was divided into five categories as (1) ≤ 5000 RMB; (2) 5001–10,000 RMB; (3) 10001–15,000 RMB; (4) > 15,000 RMB; and (5) uncertain, where 1 US$ ≈ 7.11 RMB (RMB stands for Renminbi, the official name of China’s currency). The level of family harmony was assessed with the Family Harmony Scale-5 (FHS-5), a validated questionnaire with 5 items including effective communication, conflict resolution, forbearance, family identity, and quality time with family [21]. Each item was rated from strongly disagree (score 1) to strongly agree (score 5). A total score (range: 5–25) was computed by summing the 5 items, with a higher total score indicating better family harmony. Parenting styles were assessed with the validated Chinese version of Short Egna Minnen Beträffande Uppfostran Parent Form (s-EMBU-C), which consists of 21 items and three subscales, including rejection (6 items), emotional warmth (7 items), and overprotection/control (8 items) [22]. All items were rated on a 4-point Likert scale (1 = never, 2 = seldom, 3 = often, and 4 = most of the time). The score of each type of parenting style was calculated by summing the items in the corresponding dimension. Parental negative emotional states, including depression, anxiety, and stress, were assessed by self-reported Depression Anxiety Stress Scale-21 (DASS-21) [23], a valid and reliable questionnaire in Chinese population [24]. It is consisted of 21 items that were rated based on a 4-point Likert scale (from 0 = “did not apply to me at all” to 3 = “applied to me very much, or most of the time”). The score of each negative emotion was calculated by adding up the seven items in the corresponding dimension, and higher scores indicated more negative emotions. Negative emotional states of depression, anxiety, and stress were further defined using cut-off points of 9, 7, and 14, respectively.

### Statistical analysis

Descriptive data were presented as mean (standard deviation, SD) or frequencies (%), where appropriate. Characteristics between excessive and moderate screen time groups, or between early screen exposure and later initiation of screen exposure groups, were compared by independent student t-test for continuous data and Chi-square test for categorical data. If Chi-square tests were significant for variables with more than 2 categories, post-hoc tests were further applied for multiple comparisons.

To examine the impact of excessive screen time and early screen exposure on children’s HRQOL scores and behavioral problems, linear and logistic regression models were established, respectively. For daily screen time, moderate screen time (≤ 1 h/day) was treated as the reference group, while for age of first screen use, later initiation of screen exposure (≥2 years) was used as the reference group. The associations were first assessed in crude models. The adjusted models were further controlled for children’s age, gender, status of single child, primary caregiver, parental age, marital status, education level, monthly per-capita income, family harmony level, parenting styles, and parental negative emotional states. To confirm the independent impact of excessive screen time and early screen exposure on the outcomes, these two factors were also mutually adjusted in the multivariate models. To further explore the interactive effect of excessive screen time and early screen exposure on children’s HRQOL scores and behavioral problems, we performed stratified analyses by different groups of the two exposures (i.e., 1. screen time ≤ 1 h/d and age at first screen use ≥2 years, 2. screen time ≤ 1 h/d and age at first screen use < 2 years, 3. screen time > 1 h/d and age at first screen use ≥2 years, and 4. screen time > 1 h/d and age at first screen use < 2 years) and tests for interaction. Regression models included the same confounders listed in the adjusted model, except for the two exposures.

Since parents with negative emotional states might underreport the HRQOL scores and overreport the behavioral problems of their children [25, 26], we reanalyzed the independent and interactive associations of excessive screen time and early screen exposure with the outcomes by excluding parents with negative emotional states of depression, anxiety, and stress in the sensitivity analysis.

For all linear regression models, assumptions of linear regression models, including linearity, normality, homoscedasticity, and absence of multicollinearity were checked. Beta coefficient (*β*) and corresponding 95% confidence intervals (CI) were reported by linear regression analyses, while odds ratio (OR) with 95%CI were calculated by logistic regression analyses.

Data analyses were performed using STATA/SE version 15.1. A two-sided *p* value < 0.05 was considered statistically significant.

## Results

Of the 4985 children included, 2593 (52.0%) were boys and the mean age was 4.6 (SD: 1.0) years. The average time of daily exposure to screen-based media was 0.98 (SD: 0.87) hours. Approximately 34.8% (*n* = 1734) of the children attending preschools had excessive screen time over 1 h/day, and 11.9% (*n* = 592) had started using screen-based media before the age of 2 years. Compared to children with moderate screen time, those with excessive screen time were more likely to have primary caregivers other than parents. Parents of children with excessive screen time were younger, less educated, had lower monthly per-capita income, and were more likely to adopt rejecting and overprotective parenting styles and have negative emotional states of depression, anxiety, and stress. In addition, children with early screen exposure were younger, were more likely to live in a family with low levels of family harmony, and their parents were more likely to have negative emotional states of anxiety and stress, compared to those who started using screen-based media after 2 years old (Table 1).

**Table 1:** Comparison of characteristics by children’s status of excessive screen time and early screen exposure

Characteristics	Excessive screen time		***p*** value	Early screen exposure		***p*** value
	No	Yes		No	Yes	
	***N*** = 3251	***N*** = 1734		***N*** = 4393	***N*** = 592	
**Children**						
Mean age (years), mean (SD)	4.6 (1.0)	4.6 (0.9)	0.397	4.6 (1.0)	4.5 (1.0)	< 0.001
Gender, n (%)^c^			0.514			0.434
Boy	1702 (52.4%)	891 (51.4%)		2294 (52.2%)	299 (50.5%)	
Girl	1549 (47.6%)	843 (48.6%)		2099 (47.8%)	293 (49.5%)	
Single child, n (%)^c^			0.074			0.541
Yes	1573 (48.4%)	793 (45.7%)		2092 (47.6%)	274 (46.3%)	
No	1678 (51.6%)	941 (54.3%)		2301 (52.4%)	318 (53.7%)	
Primary caregiver, n (%)^c^			< 0.001			0.049
Mother	2275 (70.0%)^a^	1117 (64.4%)^b^		3011 (68.5%)^a^	381 (64.4%)^b^	
Father	206 (6.3%)^a^	127 (7.3%)^a^		296 (6.7%)^a^	37 (6.3%)^a^	
Others	770 (23.7%)^a^	490 (28.3%)^b^		1086 (24.7%)^a^	174 (29.4%)^b^	
**Parent**						
Mather’s age, mean (SD)	33.5 (4.5)	32.6 (4.7)	< 0.001	33.2 (4.6)	33.2 (4.7)	0.933
Father’s age, mean (SD)	35.8 (5.6)	35.1 (5.5)	< 0.001	35.6 (5.5)	35.5 (5.7)	0.842
Parental marital status, n (%)^c^			0.001			0.523
Married	3131 (96.3%)	1635 (94.3%)		4203 (95.7%)	563 (95.1%)	
Unmarried	120 (3.7%)	99 (5.7%)		190 (4.3%)	29 (4.9%)	
Education level, n (%)^c^			< 0.001			0.567
Junior high school or below	246 (7.6%)^a^	208 (12.0%)^b^		394 (9.0%)	60 (10.1%)	
Senior high school	622 (19.1%)^a^	493 (28.5%)^b^		986 (22.5%)	129 (21.8%)	
Bachelor’s degree	2146 (66.1%)^a^	975 (56.3%)^b^		2758 (62.8%)	363 (61.3%)	
Master’s degree or above	235 (7.2%)^a^	56 (3.2%)^b^		251 (5.7%)	40 (6.8%)	
Monthly per-capita income, n (%)^c^			< 0.001			0.612
≤ 5000 RMB	796 (24.5%)^a^	562 (32.4%)^b^		1204 (27.4%)	154 (26.0%)	
5001–10,000 RMB	903 (27.8%)^a^	454 (26.2%)^a^		1207 (27.5%)	150 (25.3%)	
10,001–15,000 RMB	472 (14.5%)^a^	256 (14.8%)^a^		635 (14.5%)	93 (15.7%)	
> 15,000 RMB	668 (20.5%)^a^	279 (16.1%)^b^		827 (18.8%)	120 (20.3%)	
Uncertain	412 (12.7%)^a^	183 (10.6%)^b^		520 (11.8%)	75 (12.7%)	
Family harmony scores, mean (SD)	21.0 (4.8)	20.8 (4.6)	0.053	21.0 (4.7)	20.5 (4.5)	0.015
Parenting styles, mean (SD)						
Rejection scores	7.4 (1.7)	7.8 (1.8)	< 0.001	7.5 (1.7)	7.6 (1.7)	0.113
Emotional warmth scores	22.9 (4.6)	22.2 (4.5)	< 0.001	22.6 (4.7)	23.1 (4.0)	0.017
Overprotection/Control scores	15.5 (2.9)	15.8 (2.7)	< 0.001	15.6 (2.9)	15.7 (2.7)	0.284
Parental negative emotional states, n (%)^c^						
Depression	151 (4.6%)	117 (6.7%)	0.002	229 (5.2%)	39 (6.6%)	0.164
Anxiety	181 (5.6%)	157 (9.1%)	< 0.001	286 (6.5%)	52 (8.8%)	0.039
Stress	177 (5.4%)	125 (7.2%)	0.013	249 (5.7%)	53 (9.0%)	0.002

*SD* Standard Deviation. 1 US$ ≈ 7.11 RMB (RMB stands for Renminbi, the official name of China’s currency)

Each superscript letter (a or b) denotes a subset of excessive screen use categories or early screen exposure categories whose column proportions do not differ significantly from each other at the 0.05 level

^c^Column percentages

Table 2 presents the comparison of children’s HRQOL scores and behavioral problems by excessive screen time and early screen exposure groups, respectively. We found that compared to children with moderate screen time, those with excessive screen time had lower scores in all domains and summary scales of HRQOL (emotional functioning: 90.1 vs 92.2; social functioning: 75.5 vs 77.6; school functioning: 68.8 vs 71.4; physical functioning: 80.3 vs 83.4; psychosocial health summary score: 78.1 vs 80.4; and total scale score: 78.7 vs 81.2; all *p* values < 0.001), as well as more behavioral problems (conduct problems: 14.7% vs 9.1%; learning problems: 24.9% vs 15.8%; psychosomatic problems: 26.1% vs 20.4%; impulsive-hyperactive: 21.6% vs 12.4%; anxiety: 15.7% vs 12.0%; and hyperactivity index: 16.3% vs 9.7%, all *p* values < 0.001). Furthermore, compared to children with later initiation of screen exposure, those who started using screen-based media before the age of 2 years had significantly lower emotional functioning score (88.8 vs 91.8, *p* <  0.001) and psychosocial health summary score (78.6 vs 79.7, *p* = 0.005). The prevalence of behavioral problems was also significantly higher in children with early screen exposure, when compared to their counterparts who started using screen after 2 years old (conduct problems: 16.2% vs 10.4%, *p* <  0.001; learning problems: 24.2% vs 18.2%, *p* = 0.001; psychosomatic problems: 28.7% vs 21.5%, *p* < 0.001; impulsive-hyperactive: 19.9% vs 15.0%, *p* = 0.002; and hyperactivity index: 17.2% vs 11.3%, *p* < 0.001).

**Table 2:** Comparison of children’s HRQOL scores and behavioral problems by their status of excessive screen time and early screen exposure

Scales	Excessive screen time		***p*** value	Early screen exposure		***p*** value
	No	Yes		No	Yes	
	***N*** = 3251	***N*** = 1734		***N*** = 4393	***N*** = 592	
**HRQOL**, mean (SD)						
Emotional functioning	92.2 (12.5)	90.1 (14.1)	< 0.001	91.8 (12.9)	88.8 (14.5)	< 0.001
Social functioning	77.6 (11.3)	75.5 (12.1)	< 0.001	76.9 (11.6)	76.3 (12.2)	0.227
School functioning	71.4 (15.3)	68.8 (14.8)	< 0.001	70.5 (15.1)	70.6 (15.4)	0.842
Physical functioning	83.4 (20.3)	80.3 (20.3)	< 0.001	82.3 (20.5)	82.7 (19.7)	0.644
Psychosocial health summary score	80.4 (9.1)	78.1 (9.6)	< 0.001	79.7 (9.3)	78.6 (9.9)	0.005
Total scale score	81.2 (9.5)	78.7 (10.0)	< 0.001	80.4 (9.7)	79.6 (10.4)	0.078
**Behavioral problems,** n (%)						
Conduct problems	296 (9.1%)	255 (14.7%)	< 0.001	455 (10.4%)	96 (16.2%)	< 0.001
Learning problems	513 (15.8%)	431 (24.9%)	< 0.001	801 (18.2%)	143 (24.2%)	0.001
Psychosomatic problems	664 (20.4%)	452 (26.1%)	< 0.001	946 (21.5%)	170 (28.7%)	< 0.001
Impulsive-hyperactive	402 (12.4%)	375 (21.6%)	< 0.001	659 (15.0%)	118 (19.9%)	0.002
Anxiety	391 (12.0%)	273 (15.7%)	< 0.001	578 (13.2%)	86 (14.5%)	0.357
Hyperactivity index	316 (9.7%)	283 (16.3%)	< 0.001	497 (11.3%)	102 (17.2%)	< 0.001

*SD* Standard Deviation

The associations of excessive screen time and early screen exposure with children’s HRQOL scores are shown in Table 3. After adjusting for confounders, excessive screen time over 1 h/day was associated with poorer emotional functioning (*β* = − 1.30, 95%CI: − 2.02, − 0.59), social functioning (*β* = − 0.97, 95%CI: − 1.63, − 0.32), school functioning (*β* = − 1.27, 95%CI: − 2.12, − 0.43), and physical functioning (*β* = − 1.38, 95%CI: − 2.55, − 0.22). The psychosocial health summary score and total scale score were also significantly lower in children with excessive screen time, compared to their counterparts with moderate screen time (*β* = − 1.18, 95%CI: − 1.68, − 0.68 for psychosocial health summary score; *β* = − 1.23, 95%CI: − 1.76, − 0.71 for total scale score). In addition, early screen exposure before the age of 2 years was significantly associated with lower emotional functioning score (*β* = − 2.13, 95%CI: − 3.17, − 1.09), and psychosocial health summary score (*β* = − 0.82, 95%CI: − 1.54, − 0.10).

**Table 3:** Associations of excessive screen time and early screen exposure with children’s HRQOL scores

	Crude model		Adjusted model ^**a**^	
	***β*** (95%CI)	***p*** value	***β*** (95%CI)	***p*** value
**Excessive screen time**				
Emotional functioning	−2.05 (−2.81, −1.29)	< 0.001	−1.30 (−2.02, −0.59)	< 0.001
Social functioning	− 2.13 (− 2.81, − 1.46)	< 0.001	− 0.97 (− 1.63, − 0.32)	0.004
School functioning	−2.61 (−3.49, − 1.73)	< 0.001	− 1.27 (− 2.12, − 0.43)	0.003
Physical functioning	−3.16 (−4.34, − 1.97)	< 0.001	− 1.38 (− 2.55, − 0.22)	0.020
Psychosocial health summary score	−2.26 (− 2.80, − 1.72)	< 0.001	− 1.18 (− 1.68, − 0.68)	< 0.001
Total scale score	−2.49 (− 3.05, − 1.92)	< 0.001	−1.23 (− 1.76, − 0.71)	< 0.001
**Early screen exposure**				
Emotional functioning	−2.95 (− 4.07, − 1.83)	< 0.001	− 2.13 (− 3.17, − 1.09)	< 0.001
Social functioning	−0.62 (− 1.62, 0.38)	0.227	− 0.64 (− 1.59, 0.31)	0.184
School functioning	0.13 (− 1.17, 1.43)	0.842	0.31 (− 0.91, 1.53)	0.617
Physical functioning	0.41 (− 1.34, 2.16)	0.644	0.40 (− 1.28, 2.07)	0.645
Psychosocial health summary score	−1.14 (− 1.94, −0.34)	0.005	−0.82 (− 1.54, − 0.10)	0.026
Total scale score	− 0.76 (− 1.59, 0.08)	0.078	−0.52 (− 1.28, 0.24)	0.182

^a^Adjusted models were controlled for children’s age, gender, status of single child, primary caregiver, parental age, marital status, education level, monthly per-capita income, family harmony level, parenting styles, parental negative emotional states, early screen exposure (when excessive screen time was the exposure), and excessive screen time (when early screen use was the exposure)

We further evaluated the associations of excessive screen time and early screen exposure with children’s behavioral problems (Table 4). With inclusion of confounders into the models, excessive screen time was associated with increased risk of conduct problems (OR = 1.43, 95%CI: 1.17, 1.75), learning problems (OR = 1.49, 95%CI: 1.28, 1.74), psychosomatic problems (OR = 1.23, 95%CI: 1.06, 1.42), impulsive-hyperactive (OR = 1.61, 95%CI: 1.36, 1.91), anxiety (OR = 1.23, 95%CI: 1.02, 1.47), and hyperactivity index (OR = 1.49, 95%CI: 1.23, 1.80). Furthermore, compared to children who started using screen after 2 years old, those with early screen exposure were more likely to have conduct problems (OR = 1.59, 95%CI: 1.22, 2.07), learning problems (OR = 1.35, 95%CI: 1.08, 1.68), psychosomatic problems (OR = 1.41, 95%CI: 1.15, 1.72), impulsive-hyperactive (OR = 1.35, 95%CI: 1.06, 1.72), and hyperactivity index (OR = 1.57, 95%CI: 1.21, 2.02) in the multivariate models.

**Table 4:** Associations of excessive screen time and early screen exposure with children’s behavioral problems

	Crude model		Adjusted model ^**a**^	
	OR (95%CI)	***p*** value	OR (95%CI)	***p*** value
**Excessive screen time**				
Conduct problems	1.72 (1.44, 2.06)	< 0.001	1.43 (1.17, 1.75)	< 0.001
Learning problems	1.77 (1.53, 2.04)	< 0.001	1.49 (1.28, 1.74)	< 0.001
Psychosomatic problems	1.37 (1.20, 1.58)	< 0.001	1.23 (1.06, 1.42)	0.006
Impulsive-hyperactive	1.96 (1.68, 2.28)	< 0.001	1.61 (1.36, 1.91)	< 0.001
Anxiety	1.37 (1.16, 1.62)	< 0.001	1.23 (1.02, 1.47)	0.027
Hyperactivity index	1.81 (1.52, 2.15)	< 0.001	1.49 (1.23, 1.80)	< 0.001
**Early screen exposure**				
Conduct problems	1.68 (1.32, 2.13)	< 0.001	1.59 (1.22, 2.07)	0.001
Learning problems	1.43 (1.17, 1.75)	0.001	1.35 (1.08, 1.68)	0.007
Psychosomatic problems	1.47 (1.21, 1.78)	< 0.001	1.41 (1.15, 1.72)	0.001
Impulsive-hyperactive	1.41 (1.13, 1.75)	0.002	1.35 (1.06, 1.72)	0.015
Anxiety	1.12 (0.88, 1.43)	0.357	1.01 (0.78, 1.31)	0.950
Hyperactivity index	1.63 (1.29, 2.06)	< 0.001	1.57 (1.21, 2.02)	0.001

^a^Adjusted models were controlled for children’s age, gender, status of single child, primary caregiver, parental age, marital status, education level, monthly per-capita income, family harmony level, parenting styles, parental negative emotional states, early screen exposure (when excessive screen time was the exposure), and excessive screen time (when early screen use was the exposure)

Table 5 presents the interactive effect of excessive screen time and early screen exposure with HRQOL scores and behavioral problems in children. We only observed statistically significant interactive effect of excessive screen time and early screen exposure on emotional functioning domain of HRQOL and conduct problems. In sensitivity analyses, consistent results were also found after excluding parents with negative emotional states (Supplementary Tables 1–3).

**Table 5:** The interactive effect of excessive screen time and early screen exposure with children’s HRQOL scores and behavioral problems

	Screen time ≤ 1 h/d and Age at first screen use ≥ 2 years ***N*** = 2881	Screen time ≤ 1 h/d and Age at first screen use < 2 years ***N*** = 370		Screen time > 1 h/d and Age at first screen use ≥ 2 years ***N*** = 1512		Screen time > 1 h/d and Age at first screen use < 2 years ***N*** = 222		***p*** value for interaction
**HRQOL**	**Reference**	***β*** **(95%CI)**	***p*** **value**	***β*** **(95%CI)**	***p*** **value**	***β*** **(95%CI)**	***p*** **value**	
Emotional functioning	0	−2.31 (−3.62, −1.01)	0.001	−1.39 (−2.15, −0.62)	< 0.001	− 3.12 (−4.77, − 1.46)	< 0.001	< 0.05
Social functioning	0	−1.00 (− 2.21, 0.21)	0.104	− 1.28 (− 1.99, − 0.58)	< 0.001	− 0.53 (− 2.06, 0.99)	0.494	> 0.05
School functioning	0	− 0.89 (− 2.44, 0.65)	0.258	−1.50 (− 2.40, − 0.59)	0.001	0.15 (− 1.81, 2.10)	0.884	> 0.05
Physical functioning	0	0.91 (−1.23, 3.06)	0.404	−1.58 (− 2.84, − 0.32)	0.014	− 0.55 (− 3.27, 2.17)	0.692	> 0.05
Psychosocial health summary score	0	−1.40 (− 2.31, − 0.50)	0.002	− 1.39 (− 1.92, − 0.86)	< 0.001	− 1.17 (− 2.32, − 0.02)	0.046	> 0.05
Total scale score	0	− 0.82 (− 1.78, 0.14)	0.093	− 1.44 (− 2.00, − 0.87)	< 0.001	−1.01 (− 2.23, 0.20)	0.102	> 0.05
**Behavioral problems**	**Reference**	**OR (95%CI)**	***p*** **value**	**OR (95%CI)**	***p*** **value**	**OR (95%CI)**	***p*** **value**	
Conduct problems	1	1.25 (0.87, 1.82)	0.231	1.31 (1.05, 1.63)	0.015	2.77 (1.92, 4.01)	< 0.001	< 0.05
Learning problems	1	1.44 (1.08, 1.91)	0.013	1.53 (1.30, 1.81)	< 0.001	1.84 (1.32, 2.56)	< 0.001	> 0.05
Psychosomatic problems	1	1.49 (1.16, 1.93)	0.002	1.25 (1.07, 1.47)	0.004	1.60 (1.17, 2.19)	0.003	> 0.05
Impulsive-hyperactive	1	1.38 (1.00, 1.91)	0.050	1.63 (1.36, 1.96)	< 0.001	2.10 (1.47, 2.98)	< 0.001	> 0.05
Anxiety	1	1.12 (0.81, 1.57)	0.488	1.28 (1.05, 1.55)	0.014	1.08 (0.71, 1.63)	0.731	> 0.05
Hyperactivity index	1	1.63 (1.16, 2.29)	0.005	1.51 (1.23, 1.85)	< 0.001	2.26 (1.55, 3.30)	< 0.001	> 0.05

Models were controlled for children’s age, gender, status of single child, primary caregiver, parental age, marital status, education level, monthly per-capita income, family harmony level, parenting styles, and parental negative emotional states

## Discussion

In this cross-sectional study of 4985 children attending preschools, we found that excessive screen time over 1 h per day and screen exposure before the age of 2 years were two independent risk factors of lower HRQOL scores and behavioral problems. Furthermore, we observed significant interaction effect between these two factors on the emotional functioning domain of HRQOL and conduct problems.

Our findings are consistent with previous studies that have shown a significant association between excessive screen time and lower scores of HRQOL [27–29]. For example, a cross-sectional study in Hong Kong with 7555 children aged between 6 and 17 years has found that excessive screen time was negatively associated with PedsQL physical summary score, psychosocial summary score, and total scale score in both boys and girls [27]. Another cross-sectional study of children aged between 2 and 12 years in Australia has also demonstrated significant associations between excessive screen time and reduced HRQOL scores [28]. Furthermore, in line with several previous studies, we found that excessive screen time was also associated with an increased risk of behavioral problems [30, 31]. A cross-sectional analysis using data from All Our Families research has revealed a positive dose-response association between daily screen time and risk of behavioral problems among children aged 3 years [30]. Similar results have also been found in a birth cohort conducted in China, which has identified significant associations of excessive screen time in early childhood with later emotional and behavioral problems in children aged 4 years [31].

Several possible mechanisms may explain the significant associations of excessive screen time with lower HRQOL scores and increased risk of behavioral problems in children. First, increased screen time was associated with lower fractional anisotropy and higher radial diffusivity in brain white matter tracts that support executive functions, reading skills, and language development, which could further shape the well-being and behaviors in preschool children [32]. Second, according to the time displacement hypothesis, screen viewing may displace the time that could otherwise have been used for intellectually demanding activities or health-enhancing physical activities [33–35]. Lack of such activities might consequently lead to impaired HRQOL and more behavioral problems in children [36–39]. Third, the social withdrawal hypothesis has suggested that excessive screen time could hinder children’s social interaction with their peers and parents, which may subsequently jeopardize children's psychosocial well-being and prosocial behaviors [40, 41]. Fourth, children with excessive screen time usually have attention problems [42], which have been demonstrated to be associated with lower scores of HRQOL and more behavioral problems [43, 44]. Therefore, the significant associations we observed between excessive screen time and lower HRQOL scores and more behavioral problems were plausible. Nevertheless, research is still needed to further confirm the potential mechanisms.

In terms of the associations between early initiation of screen use and adverse outcomes, a case-control study with children aged between 15 to 48 months has found that compared to children who started watching television after 1 year old, those with early screen exposure were more likely to have problems in language development [9]. A longitudinal study has further demonstrated that earlier age of onset of screen exposure was associated with decreased cognitive function in preschoolers [45]. In line with these findings, our study also showed that early initiation of screen use was associated with reduced HRQOL scores and increased risk of behavioral problems among children attending preschools. One possible explanation for this association was related to the possible impairment of brain development during childhood caused by early initiation of screen use. It is well-recognized that the period from birth to 2 years of age is important for cognition establishment and behaviors that last a lifetime [46]. During this period, cortical grey matter undergoes robust development, along with rapid myelination and maturation of the microstructure of existing white-matter tracts [47–51]. When children exposed to screen-based media before 2 years, their normal development of brain network might be interrupted with neurochemical and anatomical brain changes, and eventually lead to poor HRQOL and behavioral problems. However, future studies are warranted to investigate the mechanisms.

The present study further extends the findings of existing literatures by showing the independent role of excessive screen time and early screen exposure on children’s HRQOL and behavioral problems. We additionally found significant interactive effect of the two exposures on children’s emotional functioning domain of HRQOL and conduct problems, but not for other domains. One possible explanation might due to the small sample size of children with both excessive screen time and early screen exposure (*N* = 222), resulting in low statistical power. Another possible explanation might be related to the cross-sectional nature of this study. The daily time spent on screen might have changed since they first had access to screen-based media, which cannot be captured by current study design. Additional research with repeated data from follow-up studies is needed to further investigate the interactive role of excessive screen time and early exposure on health outcomes.

Although our study has included a large sample size and adjusted for several potential confounders, there are still some limitations that deserve discussion. First, the cross-sectional design precludes us from confirming the causal relationship. Also, we were unable to evaluate the moderating role of age in the associations between excessive screen use and early screen exposure with HRQOL scores and behavioral problems, as we cannot ensure that whether children have changed their screen-usage patterns. Further well-designed longitudinal studies with accumulated data are needed to clarify the temporal associations of screen use habits with HRQOL and behaviors in children attending preschools. Second, while self-reporting of screen use, HRQOL, and behaviors are more accurate, it should be noted that these data were measured by proxy because of children’s young age, which is a common limitation faced by child health studies [52, 53]. Third, studies have demonstrated that high-quality programming and parents’ co-viewing with children were beneficial to children’s social skills, sleep patterns, and prosocial behaviors [54–56]. Nevertheless, our study did not collect these data. Further research is needed to explore the associations of co-viewing high-quality programming with children’s HRQOL and behavioral problems. Fourth, inconsistent findings have been reported between the types of devices used and children’s health outcomes [57, 58]. We were unable to illustrate on this issue due to data unavailability. In addition, although previous studies have suggested that background exposure of screen use (i.e., with screen on, but no one is watching) was associated with adverse health outcomes in children, such as decreased language skills and executive function [56, 59], we did not take it into consideration in the current study. The associations of background exposure and device-specific screen use with children’s outcomes should be further elucidated in future studies. Last, although we have adjusted for several confounders in the analysis, we could not rule out the possibility of residual confounding effects that may distort the associations, such as children’s temperament, parental physical or mental illness, and parents’ attitude towards children’s screen use [52, 53, 60–62].

## Conclusion

In conclusion, excessive screen time and early screen exposure were found to have independent and interactive effects on poor HRQOL and behavioral problems in children attending preschools after adjusting for confounding factors. Our findings support current guidelines regarding appropriate screen use in children. The study also implicates that avoiding screen use during infancy and reducing daily screen time may preserve HRQOL and prevent behavioral problems in children attending preschools. However, future longitudinal studies are needed to confirm the conclusions.

## Supplementary Information

**Additional file 1: Supplementary Table 1.** Associations of excessive screen time and early screen exposure with children's HRQOL scores, excluding parents with negative emotional states. **Supplementary Table 2.** Associations of excessive screen time and early screen exposure with children's behavioral problems, excluding parents with negative emotional states. **Supplementary Table 3.** The interactive effect of excessive screen time and early screen exposure with children's HRQOL scores and behavioral problems, excluding parents with negative emotional states.

## Data Availability

The datasets used and analyzed during the current study are not publicly available for ethical and privacy reasons but are available from the corresponding author upon reasonable request.
