# Supplementary material for: Associations of excessive screen time and early screen exposure with health-related quality of life and behavioral problems among children attending preschools
Source: BMC Public Health. 2022 Dec 27;22:2440. doi: 10.1186/s12889-022-14910-2 (PMC9795659; doi:10.1186/s12889-022-14910-2)
Supplement: Supplementary file 1 — Additional file 1: Supplementary Table 1. Associations of excessive screen time and early screen exposure with children's HRQOL scores, excluding parents with negative emotional states. Supplementary Table 2. Associations of excessive screen time and early screen exposure with children's behavioral problems, excluding parents with negative emotional states. Supplementary Table 3. The interactive effect of excessive screen time and early screen exposure with children's HRQOL scores and behavioral problems, excluding parents with negative emotional states. [file 12889_2022_14910_MOESM1_ESM.docx]

**Supplementary Table 1 Associations of excessive screen time and early screen exposure with children’s HRQOL scores, excluding parents with negative emotional states**

|  |  | **Crude model** | |  | **Adjusted model ^a^** | |
| --- | --- | --- | --- | --- | --- | --- |
|  |  | ***β* (95%CI)** | ***p* value** |  | ***β* (95%CI)** | ***p* value** |
| **Excessive screen time** | |  |  |  |  |  |
|  | Emotional functioning | -1.63 (-2.37, -0.90) | <0.001 |  | -1.36 (-2.09, -0.64) | <0.001 |
|  | Social functioning | -1.55 (-2.25, -0.85) | <0.001 |  | -0.67 (-1.35, 0.02) | 0.056 |
|  | School functioning | -2.43 (-3.37, -1.49) | <0.001 |  | -1.24 (-2.14, -0.34) | 0.007 |
|  | Physical functioning | -2.33 (-3.59, -1.07) | <0.001 |  | -0.85 (-2.09, 0.39) | 0.180 |
|  | Psychosocial health summary score | -1.87 (-2.42, -1.33) | <0.001 |  | -1.09 (-1.61, -0.57) | <0.001 |
|  | Total scale score | -1.99 (-2.56, -1.41) | <0.001 |  | -1.03 (-1.58, -0.48) | <0.001 |
|  |  |  |  |  |  |  |
| **Early screen exposure** | |  |  |  |  |  |
|  | Emotional functioning | -2.43 (-3.52, -1.34) | <0.001 |  | -2.09 (-3.15, -1.04) | <0.001 |
|  | Social functioning | -0.50 (-1.55, 0.54) | 0.347 |  | -0.76 (-1.76, 0.24) | 0.139 |
|  | School functioning | 0.11 (-1.28, 1.51) | 0.875 |  | 0.21 (-1.10, 1.53) | 0.752 |
|  | Physical functioning | 1.04 (-0.83, 2.91) | 0.276 |  | 0.74 (-1.07, 2.55) | 0.423 |
|  | Psychosocial health summary score | -0.94 (-1.75, -0.13) | 0.024 |  | -0.88 (-1.64, -0.12) | 0.023 |
|  | Total scale score | -0.44 (-1.30, 0.41) | 0.310 |  | -0.47 (-1.27, 0.33) | 0.245 |

^a^ Adjusted models were controlled for children’s age, gender, status of single child, primary caregiver, parental age, marital status, education level, monthly per-capita income, family harmony level, parenting styles, early screen exposure (when excessive screen time was the exposure), and excessive screen time (when early screen use was the exposure).

**Supplementary Table 2 Associations of excessive screen time and early screen exposure with children’s behavioral problems, excluding parents with negative emotional states**

|  |  | **Crude model** | |  | **Adjusted model ^a^** | |
| --- | --- | --- | --- | --- | --- | --- |
|  |  | **OR (95%CI)** | ***p* value** |  | **AOR (95%CI)** | ***p* value** |
| **Excessive screen time** | |  |  |  |  |  |
|  | Conduct problems | 1.81 (1.46, 2.24) | <0.001 |  | 1.57 (1.25, 1.97) | <0.001 |
|  | Learning problems | 1.75 (1.49, 2.06) | <0.001 |  | 1.52 (1.28, 1.80) | <0.001 |
|  | Psychosomatic problems | 1.29 (1.11, 1.50) | 0.001 |  | 1.20 (1.03, 1.41) | 0.021 |
|  | Impulsive-hyperactive | 1.92 (1.61, 2.29) | <0.001 |  | 1.61 (1.34, 1.95) | <0.001 |
|  | Anxiety | 1.34 (1.10, 1.63) | 0.003 |  | 1.28 (1.04, 1.57) | 0.018 |
|  | Hyperactivity index | 1.88 (1.53, 2.30) | <0.001 |  | 1.58 (1.27, 1.96) | <0.001 |
|  |  |  |  |  |  |  |
| **Early screen exposure** | |  |  |  |  |  |
|  | Conduct problems | 1.78 (1.34, 2.36) | <0.001 |  | 1.71 (1.27, 2.30) | <0.001 |
|  | Learning problems | 1.47 (1.17, 1.85) | 0.001 |  | 1.44 (1.13, 1.83) | 0.003 |
|  | Psychosomatic problems | 1.49 (1.20, 1.84) | <0.001 |  | 1.45 (1.17, 1.80) | 0.001 |
|  | Impulsive-hyperactive | 1.43 (1.12, 1.84) | 0.005 |  | 1.42 (1.09, 1.85) | 0.010 |
|  | Anxiety | 0.98 (0.73, 1.33) | 0.915 |  | 0.95 (0.70, 1.30) | 0.761 |
|  | Hyperactivity index | 1.68 (1.28, 2.21) | <0.001 |  | 1.65 (1.23, 2.20) | 0.001 |

^a^ Adjusted models were controlled for children’s age, gender, status of single child, primary caregiver, parental age, marital status, education level, monthly per-capita income, family harmony level, parenting styles, early screen exposure (when excessive screen time was the exposure), and excessive screen time (when early screen use was the exposure).

**Supplementary Table 3 The** **interactive effect of excessive screen time and early screen exposure with children’s HRQOL scores and behavioral problems, excluding parents with negative emotional states**

|  |  | **Screen time** ≤ **1 h/d and Age at first screen use** ≥ **2 years**  **N = 2643** | | **Screen time** ≤ **1 h/d and Age**  **at first screen use < 2 years**  **N = 323** | | **Screen time >1 h/d and Age**  **at first screen use** ≥ **2 years**  **N = 1317** | | **Screen time > 1 h/d and Age**  **at first screen use < 2 years**  **N = 188** | |  | ***p* value for interaction** |
| --- | --- | --- | --- | --- | --- | --- | --- | --- | --- | --- | --- |
|  |  |  |  |  |  |  |  |  |  |  |  |
| **HRQOL** | | **Reference** | ***β* (95%CI)** | | ***p* value** | ***β* (95%CI)** | ***p* value** | ***β* (95%CI)** | ***p* value** |  |  |
|  | Emotional functioning | 0 | -2.18 (-3.50, -0.86) | | 0.001 | -1.42 (-2.18, -0.65) | <0.001 | -3.25 (-4.95, -1.56) | <0.001 |  | < 0.05 |
|  | Social functioning | 0 | -1.19 (-2.46, 0.08) | | 0.065 | -1.02 (-1.76, -0.28) | 0.007 | -0.09 (-1.72, 1.54) | 0.916 |  | > 0.05 |
|  | School functioning | 0 | -0.87 (-2.53, 0.78) | | 0.301 | -1.40 (-2.37, -0.44) | 0.004 | -0.15 (-2.27, 1.98) | 0.893 |  | > 0.05 |
|  | Physical functioning | 0 | 1.12 (-1.18, 3.43) | | 0.338 | -1.13 (-2.47, 0.21) | 0.098 | 0.73 (-2.23, 3.69) | 0.629 |  | > 0.05 |
|  | Psychosocial health summary score | 0 | -1.41 (-2.36, -0.47) | | 0.003 | -1.28 (-1.83, -0.73) | <0.001 | -1.16 (-2.38, 0.05) | 0.061 |  | > 0.05 |
|  | Total scale score | 0 | -0.78 (-1.79, 0.23) | | 0.129 | -0.69 (-1.98, 0.60) | <0.001 | -1.24 (-1.83, -0.66) | 0.296 |  | > 0.05 |
| **Behavioral problems** | | **Reference** | **OR (95%CI)** | | ***p* value** | **OR (95%CI)** | ***p* value** | **OR (95%CI)** | ***p* value** |  |  |
|  | Conduct problems | 1 | 1.34 (0.88, 2.06) | | 0.177 | 1.42 (1.11, 1.82) | 0.006 | 3.25 (2.16, 4.90) | <0.001 |  | < 0.05 |
|  | Learning problems | 1 | 1.47 (1.08, 2.02) | | 0.016 | 1.54 (1.28, 1.85) | <0.001 | 2.07 (1.45, 2.97) | <0.001 |  | > 0.05 |
|  | Psychosomatic problems | 1 | 1.53 (1.17, 2.01) | | 0.002 | 1.23 (1.04, 1.45) | 0.018 | 1.64 (1.16, 2.31) | 0.005 |  | > 0.05 |
|  | Impulsive-hyperactive | 1 | 1.49 (1.05, 2.12) | | 0.027 | 1.65 (1.35, 2.02) | <0.001 | 2.15 (1.45, 3.18) | <0.001 |  | > 0.05 |
|  | Anxiety | 1 | 1.05 (0.72, 1.55) | | 0.791 | 1.32 (1.06, 1.64) | 0.012 | 1.07 (0.65, 1.75) | 0.785 |  | > 0.05 |
|  | Hyperactivity index | 1 | 1.72 (1.17, 2.54) | | 0.006 | 1.60 (1.26, 2.02) | <0.001 | 2.53 (1.66, 3.85) | <0.001 |  | > 0.05 |

Models were controlled for children’s age, gender, status of single child, primary caregiver, parental age, marital status, education level, monthly per-capita income, family harmony level, and parenting styles.
